# Rapid *in situ* assessment of Cu-ion mediated effects and antibacterial efficacy of copper surfaces

**DOI:** 10.1038/s41598-018-26391-8

**Published:** 2018-05-25

**Authors:** Merilin Rosenberg, Heiki Vija, Anne Kahru, C. William Keevil, Angela Ivask

**Affiliations:** 1 Laboratory of Environmental Toxicology, National Institute of Chemical Physics and Biophysics, Tallinn, Estonia; 2 Department of Natural Sciences, Tallinn University of Technology, Tallinn, Estonia; 3 Estonian Academy of Sciences, Kohtu 6, Tallinn, Estonia; 4 Faculty of Natural and Environmental Sciences, Centre for Biological Sciences, University of Southampton, Southampton, UK

## Abstract

Release of metal ions from metal-based surfaces has been considered one of the main drivers of their antimicrobial activity. Here we describe a method that enables parallel assessment of metal ion release from solid metallic surfaces and antimicrobial efficacy of these surfaces in a short time period. The protocol involves placement of a small volume of bioluminescent bacteria onto the tested surface and direct measurement of bioluminescence at various time points. In this study, two recombinant *Escherichia coli* strains, one expressing bioluminescence constitutively and applicable for general antimicrobial testing, and the other induced by Cu ions, were selected. Decrease in bioluminescence of constitutive *E*. *coli* on the surfaces showed a good correlation with the decrease in bacterial viability. Response of Cu-inducible *E*. *coli* showed a correlation with Cu content in the tested surfaces but not with Cu dissolution suggesting the role of direct bacteria-surface contact in Cu ion-driven antibacterial effects. In summary, the presented protocol enables the analysis of microbial toxicity and bioavailability of surface-released metal ions directly on solid surfaces within 30–60 min. Although optimized for copper and copper alloy surfaces and *E*. *coli*, the method can be extended to other types of metallic surfaces and bacterial strains.

## Introduction

Metal-, metal alloy or metal nanoparticle-based surfaces have been used extensively as antimicrobial surfaces in water treatment (filters, water pipes), in ventilation and air-conditioning systems, general high touch surfaces such as door handles and knobs, and more importantly in healthcare settings^1–4^. Copper-based surfaces were the first registered solid antimicrobial surfaces by US EPA in 2008, after being supported by extensive antimicrobial efficacy testing. According to those studies, copper and copper alloys such as brass and bronze were able to decrease the counts of bacteria, including pathogens such as methicillin resistant *Staphylococcus aureus*, by 7 to 8 logs within hours^5^. Remarkably, such a decrease has not only been reported in laboratory conditions but also in hospitals, in field conditions^6^. On the other hand, some studies have shown that the antimicrobial efficacy of copper-based surfaces is modest and statistically not sound in preventing healthcare associated infections and spreading of antibiotic resistant organisms^2^. It is likely that these discrepancies between the different test results are mainly due to different testing conditions. Indeed, antimicrobial activity of copper surfaces has been shown to be affected by test conditions such as temperature and relative humidity^4,7–10^ as well as by the presence of any residues, or for example an oxide layer on the surfaces^11^. Thus, one step closer to obtaining relevant correct and sound information on antimicrobial efficacy of copper (and other solid) surfaces is the use of standardized and appropriate test methods. Currently, a variety of test methods and testing suggestions that differ in their requirements for inoculum volume to surface area ratio, microbial culture density, relative humidity conditions, and that require testing in wet or dry conditions, are available for solid surfaces. The standard test methods generally approved and frequently used for antimicrobial efficacy assessment in Europe, US and Japan^12–15^ usually require the placement of a small volume of microbial suspension onto the tested surface, incubation in wet or dry conditions during a pre-determined time, and subsequent assessment of microbial viability, 24–48 h after agar plating. Although testing in “dry” conditions may be closer to real environmental conditions^5,16^ several studies have shown that this test format is more prone to errors due toot experimental variations e.g., in humidity and temperature^9^ which suggests that testing in “wet” conditions may be more reliable and needs less optimization for screening purposes.

In this study, we introduce a new robust “wet” test format that can be used for the testing of solid metal-based antimicrobial surfaces and optimized this method for copper and copper alloy surfaces. The test is based on bioluminescent microbial cells, recombinant bioluminescent *Escherichia coli* cells used in this study. Due to the fact that release of copper ions is generally considered as the key mechanism of antimicrobial activity of copper surfaces^4,17^ a recombinant bioluminescent *E*. *coli* strain that enables the quantitative assessment of bioavailable Cu ions release is used in the same test format in parallel to the constitutively bioluminescent *E*. *coli*. The combination of those two bacterial strains allows the assessment released bioavailable copper from the surface and its bactericidal effect. The measurement of bioluminescence is carried out directly on surfaces and the duration of the new test format can be varied but we propose test times from 15 to 60 minutes. Although the method was optimized for copper surfaces in this study, it could be easily adapted for the assessment of metal ion release and antibacterial properties of other metal-based solid surfaces. Additionally, a constitutively bioluminescent *E*. *coli* strain can be individually used as a rapid first tool to evaluate the antibacterial effect of other (non-metallic) solid surfaces.

## Results

### Optimization of the measurement set-up

#### Microplate configuration

**Figure 1:**
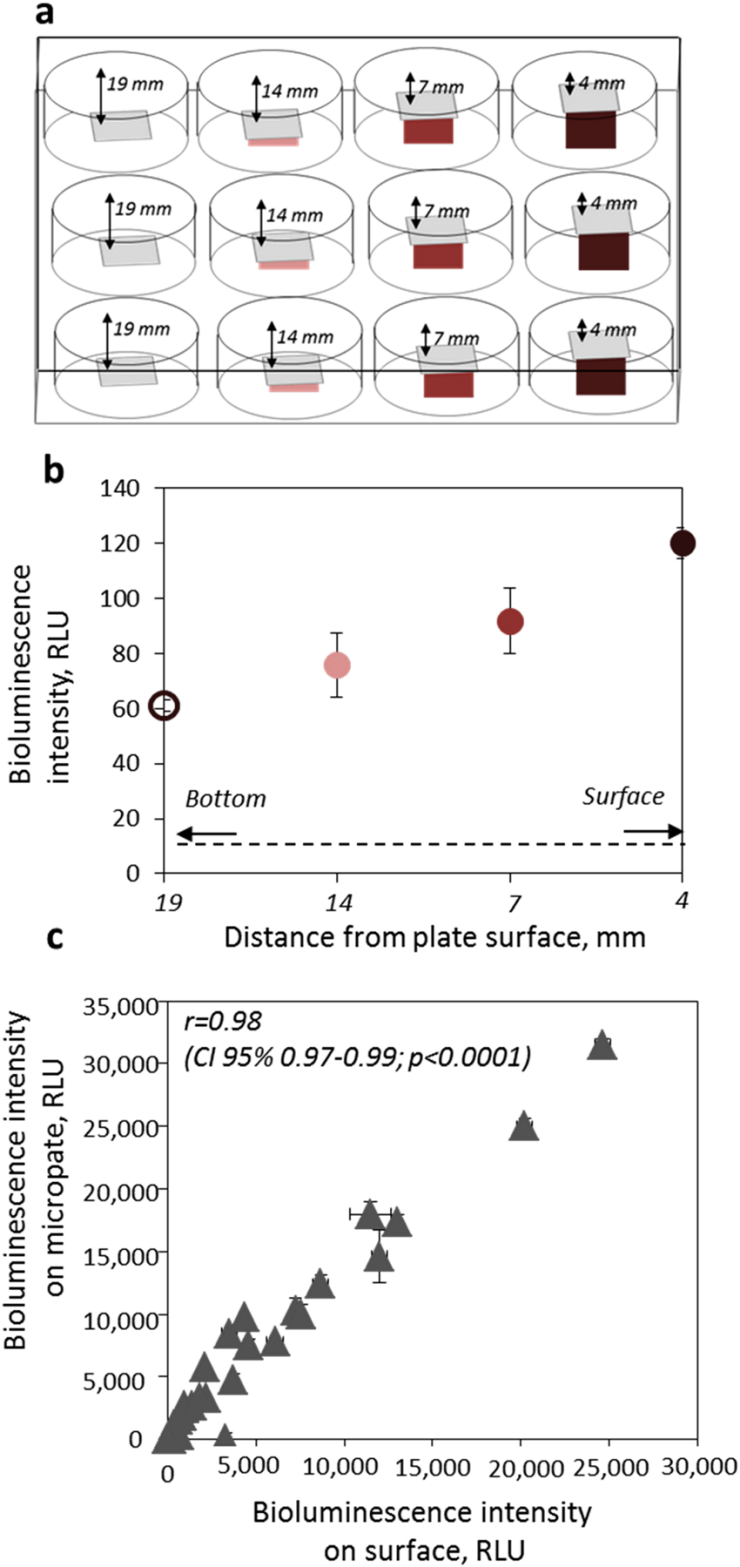
Set-up of 12-well microplate for optimal signal collection from bioluminescent bacteria that were placed onto a 1 × 1 cm copper coupon. (**a**) Is a schematic representation of 12-well microplate in which column 1 represents coupons on the plate bottom, column 2 coupons raised using 5 mm adapters, column 3 coupons raised using 12 mm and column 4 coupons raised using 15 mm adapters; (**b**) shows the measured baseline bioluminescence values of Cu biosensor *E*. *coli* (pSLcueR/pDNPcopAlux) on coupons on the plate bottom, 14, 7 and 4 mm from the plate surface. (**c**) Shows the linear correlation between bioluminescence of the Cu biosensor bacteria that had been previously exposed to known concentrations of Cu ions (0.1–100 µg Cu/mL; to cover different bioluminescence intensities) and either placed onto solid polyethylene surfaces in 12-well plates or onto the wells or 96-well microplates. Mean and standard deviation of three experiments are shown.

The novel set-up of our measurement system involving direct measurement of bacterial bioluminescence on solid surfaces was optimized to provide a reproducible test protocol. Due to the size of copper coupons (1 × 1 cm), 12-well plates were selected as a testing platform in this study. However, other plate types accommodating the surfaces of interest can also be selected. Due to the leakage of luminescence between wells in transparent plates and high background luminescence of white plates, we selected black plates from Cellvis (P12–1.5H-N). Because of the sensitivity limits of the luminometers and the fact that the bioluminescence intensity of bacteria on copper surfaces may be relatively low, we introduced adapters that increased the height of metal coupons by 5, 12 and 15 mm, respectively, to be closer to the photomultipliers collecting and amplifying the luminescence signal (Fig. 1a,b). To demonstrate the effect of adapters in obtained readings, we placed 1 × 1 cm polyethylene (PE) surfaces onto the adapters, added 75 µL suspension of bioluminescent *E*. *coli* (copper-induced strain of *E*. *coli* (pSLcueR/pDNPcopAlux) was used) and registered the baseline bioluminescence of the Cu-inducible *E*. *coli* strain. The measured values were clearly dependent on the position of the surface on the microplate wells and the highest bioluminescence reading was measured when the surfaces were lifted by 15 mm (4 mm from the surface of the microplate top) (Fig. 1b). Thus, this configuration was used in all further experiments.

**Figure 2:**
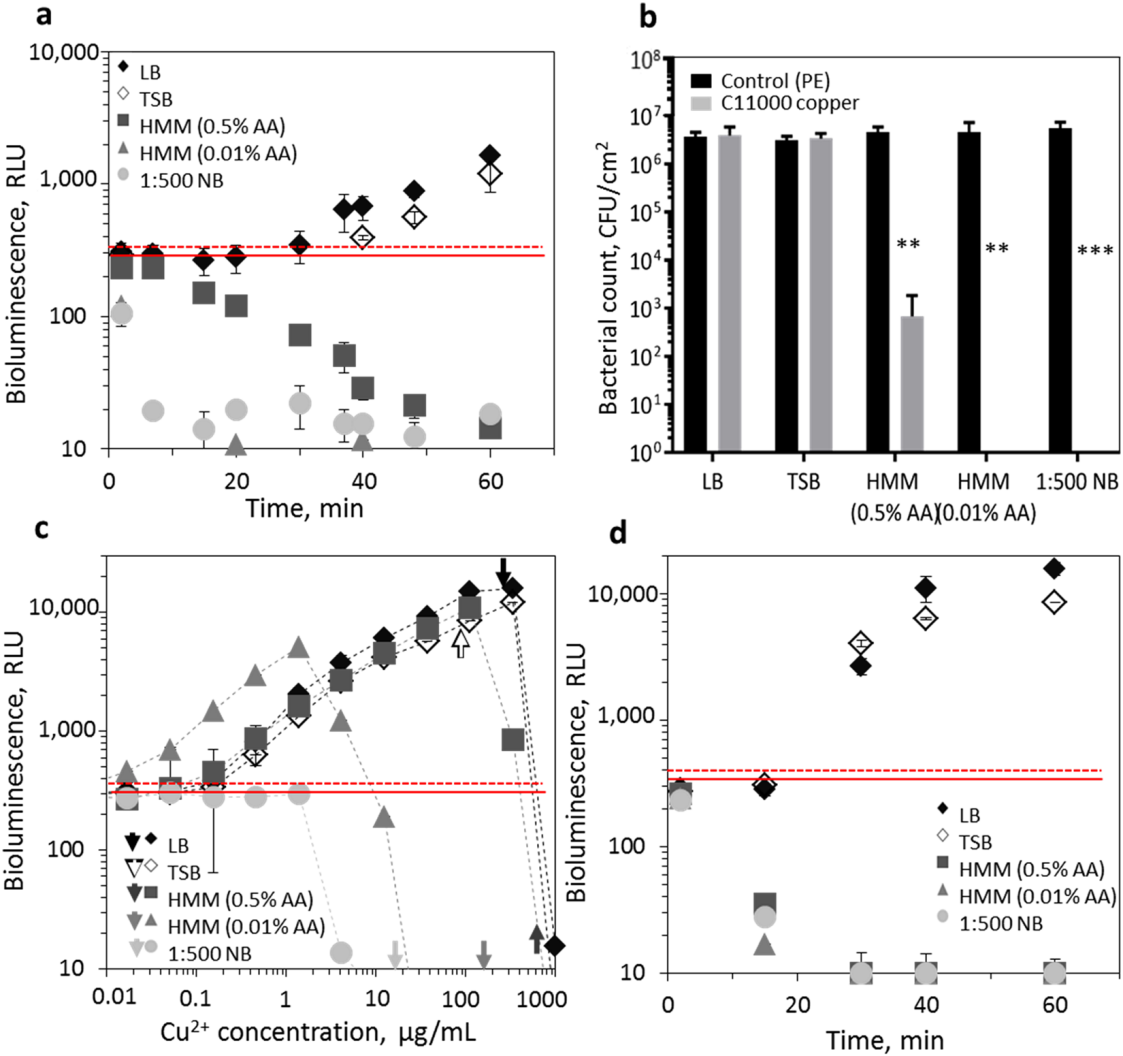
Induction of bioluminescence in *E*. *coli* (pSLcueR/pDNPcopAlux) Cu biosensor in different test media. (**a**) Bioluminescence of the Cu biosensor on C11000 copper surfaces in Heavy Metal MOPS Medium (HMM) with 0.01 and 0.5% casein acid hydrolysate (HMM 0.5% AA and 0.01% AA, respectively), 1:500 diluted Nutrient Broth (NB) medium, Luria Bertani (LB) medium and Tryptone Soy Broth (TSB) medium. (**b**) Bacterial counts after 60 min exposure in the specified media on control or C11000 surface. Significant differences in viable counts from control value based on two-way analysis of variance (ANOVA) and Bonferroni’s multiple comparisons test are marked p < 0.1(.); p < 0.05(*); p < 0.01(**); p < 0.001(***); p < 0.0001(****); (**c**) bioluminescence of the Cu biosensor in different media in CuSO_4_ standard solutions (0.01–1000 mg/L Cu^2+^) after 60 min exposure. Solid red line indicates background, i.e., non-induced bioluminescence of *E*. *coli* (pSLcueR/pDNPcopAlux) and dotted red line indicates the limit for significantly increase bioluminescence. Arrows indicate concentrations of Cu ions that according to TR-XRF were released from C11000 surfaces within 60 min in each medium. (**d**) time-dependent induction of bioluminescence in *E*. *coli* (pSLcueR/pDNPcopAlux) copper sensor in the presence of Cu concentrations indicated with arrows on (**c**). Mean and standard deviation of three replicates are shown.

In order to validate the measurement of bacterial bioluminescence on surfaces in a 12-well microplate set-up, we compared the bioluminescence values measured with the new 12-well set-up with those from an already established 96-well set-up. For that, *E*. *coli* (pSLcueR/pDNPcopAlux) was mixed with known concentrations of Cu ions which enabled us to obtain bioluminescence values at different intensities due to the fact that the bioluminescence in the copper sensor strain is induced concentration-dependently by bioavailable Cu^2+^ ions. *E*. *coli* (pSLcueR/pDNPcopAlux) mixed with various ionic copper concentrations were placed either onto PE control surfaces fitted into 12-well plate or into the wells of a 96-well plate. As seen from Fig. 2, the results obtained on 12- and 96-well microplates were in good correlation (r = 0.98; CI 95% 0.97–0.99; p < 0.0001) indicating that measurement of bioluminescence directly on solid surfaces is a viable analysis strategy.

#### Test medium

Based on information from our previous studies^18^ and the studies of other authors^19,20^ we assumed that the response of bioluminescent copper sensor *E*. *coli* (pSLcueR/pDNPcopAlux) and constitutively bioluminescent *E*. *coli* (pDNlux) is significantly affected by the test medium in which the bacteria are applied to the surfaces. As an example, Molteni *et al*.^20^ indicated that Tris-buffer dramatically enhances contact killing of bacteria on copper surfaces, due to increasing release of Cu ions in this environment compared with water or phosphate buffer. On the other hand, our previous study by Käkinen *et al*.^18^ showed that increasing organic matter decreases the toxicity of Cu formulations. Here we tested the effect of four different exposure media on bioluminescence of *E*. *coli* copper sensor bacteria on C11000 copper surfaces. We chose 1:500 diluted nutrient broth (NB) medium which is a suggested medium in the ISO 22196 standard test protocol for solid surfaces^14^; heavy metal MOPS medium (HMM) that due to its non-complexing nature^21^ has been used in a series studies focusing on metal testing (the medium was supplemented with either 0.5 or 0.01% cas-aminoacids as in^18^); and nutrient rich media LB and TSB, the first being a standard microbiological medium and the second being widely used in standard protocols, e.g., US EPA surface testing guidelines^12,13^. As seen from Fig. 2a, when applied onto C11000 surfaces, Cu biosensor bacteria *E*. *coli* (pSLcueR/pDNPcopAlux) were only induced in LB and TSB media whereas in all other tested media the bacterial bioluminescence decreased below baseline luminescence indicating the antibacterial effect of copper surfaces in these conditions. The latter was also confirmed by counting surviving bacterial cells after their 60 min exposure on copper surfaces: around 3-log reduction of Cu biosensor bacteria was observed in HMM medium supplemented with 0.5% cas-aminoacids and no viable bacteria were registered when they were exposed to copper surfaces in 0.01% cas-aminoacid supplemented HMM or in 1:500 diluted NB medium (Fig. 2b). As the release of Cu ions from copper surfaces has been considered the main reason for the toxicity of those surfaces, we assumed that the differential response of *E*. *coli* copper sensor to C11000 surfaces in the selected test media was likely due to different susceptibility of the bacteria to Cu^2+^ in those media. Therefore, we next studied the response of *E*. *coli* (pSLcueR/pDNPcopAlux) to a range of concentrations of ionic Cu which covered those released from C11000 surfaces during the testing (Fig. 2c). It was evident that HMM supplemented with 0.01% amino acids and 1:500 NB media did not support the induction of bacterial bioluminescence during 60 min exposure, likely due to relatively high toxicity of Cu ions in those media as shown by us earlier^18^ but also due to shortage of nutrients to support bacterial bioluminescence production. The best bioluminescence induction and lowest Cu ions toxicity was observed in LB and TSB where the bioluminescence inhibition of *E*. *coli* (pSLcueR/pDNPcopAlux) was measured only at ~500 µg ionic Cu/L. Comparison of the induction profiles of the *E*. *coli* Cu sensor (Fig. 2c) with the release of Cu ions from C11000 surfaces in the different media (9.8 µg Cu ions was released from one C11000 coupon in LB medium, 53 µg in HMM medium supplemented with 0.5% cas-aminoacids, 13 µg in HMM medium with 0.01% cas-aminoacids, 7 µg in TSB medium and 0.7 µg in 1:500 diluted NB medium; these concentrations in µg/mL are indicated with arrows in Fig. 2c) showed that in HMM media and in 1:500 diluted NB medium the released amount of ionic Cu was already toxic to bacteria and inhibited their bioluminescence even after 15 min exposure (Fig. 2d). However, the amount of Cu ions released from C11000 coupon in LB and TSB media was still within the measurable range in these specific media (Fig. 2c,d). Based on these results and keeping in mind the fact that one of the goals was to observe and quantitatively measure the bioluminescence induction of Cu biosensors, we selected LB medium for further testing. Although poorer media with less organic constituents would be certainly more relevant in mimicking actual exposure scenarios, Cu biosensor bacteria would not survive in these conditions with no results obtained.

#### Volume of biosensor bacteria on the surfaces

**Figure 3:**
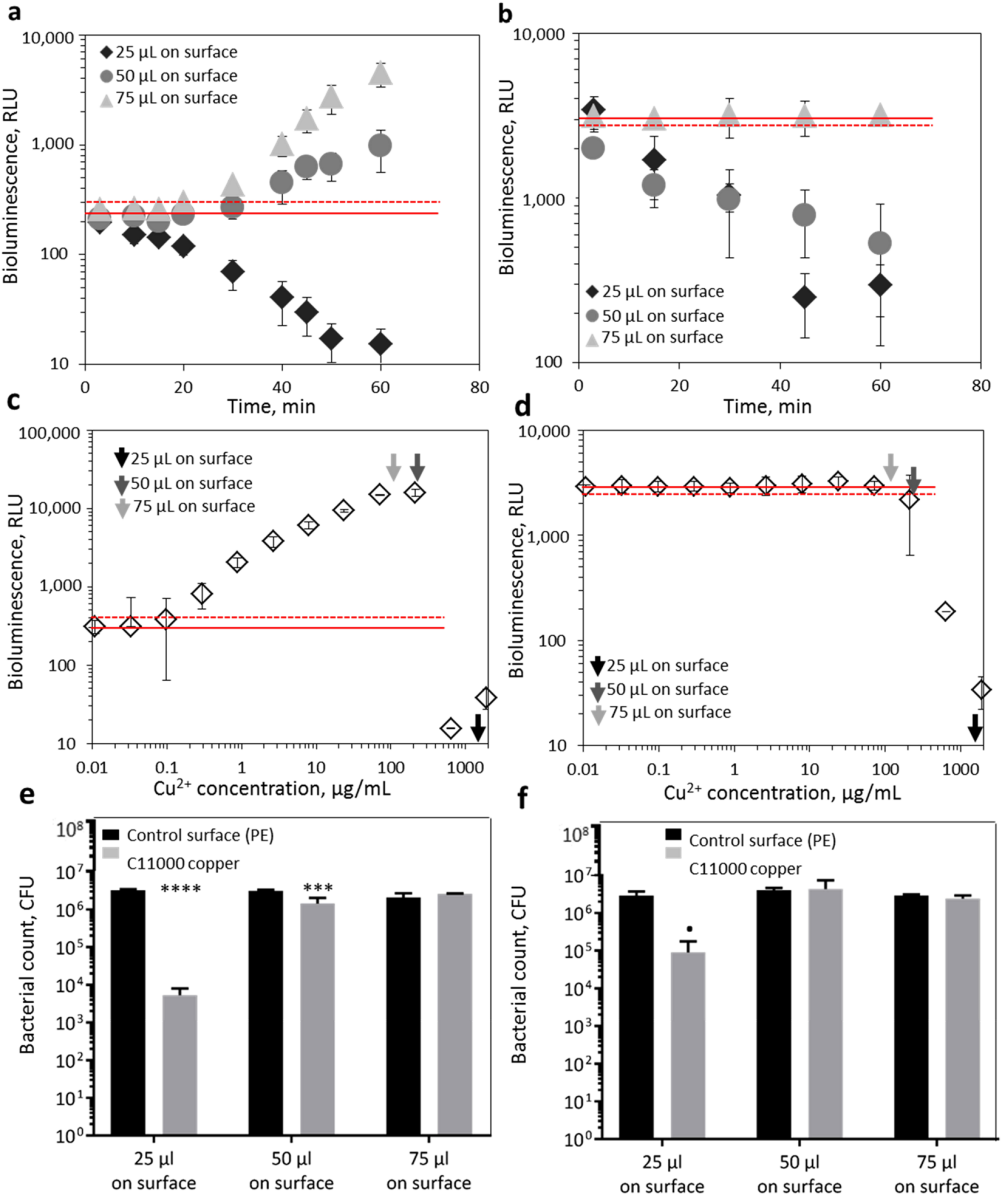
Optimization of bacterial test volume on copper surfaces. ~1 × 10^7^ cells of *E*. *coli* Cu biosensor (**a,c,e**) or constitutively bioluminescent *E*. *coli* (**b,d,f**) in 25, 50 or 75 µL was placed onto 1 × 1 cm C11000 surfaces. Bacterial bioluminescence was registered during 60 min (**a,b**). The concentration of released copper from C11000 surfaces during 60 min in 25, 50 or 75 µL (indicated with arrows) was compared with standard response curve of the Cu sensor strain and constitutively bioluminescent strain (**c,d**). The number of viable cells in 25, 50 or 75 µL Cu biosensor or constitutively bioluminescent *E*. *coli* strain on C11000 surfaces after 60 min (**e** and **f**). Significant differences in viable counts from control value based on two-way analysis of variance (ANOVA) and Bonferroni’s multiple comparisons test are marked p < 0.1(.); p < 0.05(*); p < 0.01(**); p < 0.001(***); p < 0.0001(****). Solid red dotted line designates the background luminescence of *E*. *coli* Cu biosensor or constitutively bioluminescent *E*. *coli;* dotted red line designates significant increase (*E*. *coli* Cu biosensor) or decrease (constitutively bioluminescent *E*. *coli)* of bioluminescence. Mean and standard deviation of three replicates are shown.

Due to the different requirements for bacterial volume on solid surfaces in different standard test methods (1.5 × 10^4^ cells/cm^2^ applied in 25 µL/cm^2^ under film in ISO 22196^14^; 1.6 × 10^6^ cells/cm^2^ applied in 16 µL/cm^2^ and dried in CSN EN 13697^15^; 1.4–5.8 µL/cm2 with at least 3.1 × 10^3^ recoverable CFU/cm^2^ in US EPA standards for copper surfaces^12,13^) we optimized the volume and number of bacteria on copper surfaces. It must be noted that the use of bioluminescent bacteria sets certain requirements for the assay. Specifically, the test needs to be conducted in wet conditions (i.e., measurable bioluminescence is not induced under dry conditions) and in case of copper induced bacteria, exposure times longer than 20 min are required (for bioluminescence to be induced, see Fig. 2d). Our results showed that volumes smaller than 25 µL spread on 1 cm2 (1 × 1 cm) solid coupons tended to significantly dry during 20–60 min exposure and thus, 25 µL was used as smallest volume of bacterial suspension in our optimization study. 50 µL/cm^2^ and 75 µL/cm^2^ of bacterial suspension were tested as larger volumes and, in all tested volumes, the bacterial number per surface area was kept constant (2–3 × 10^6^ cells/cm^2^). As seen from Fig. 3, when 25 µL of *E*. *coli* (pSLcueR/pDNPcopAlux) copper sensor bacteria was applied to and spread on a C11000 copper coupon, the bioluminescence decreased after 10 min (Fig. 3a). A similar time-dependent decrease in bioluminescence was observed in case of constitutively bioluminescent *E*. *coli* (pDNlux) (Fig. 3b). This decrease in bioluminescence was likely due the release of high concentrations of Cu ions from the surfaces in the 25 µL volume (30 µg Cu released per 1 cm^2^ Cu surface, i.e., bacteria on the coupon surface were exposed to 1200 µg Cu/mL). Indeed, when the released concentration of Cu was compared with the response of bioluminescent bacteria to copper standard solutions, it was obvious that it was already toxic, both to *E*. *coli* copper sensor as well as to constitutively bioluminescent *E*. *coli* (see black arrows in Fig. 3c,d). However, drying of 25 µL bacterial suspension on copper surfaces could have also contributed to bioluminescence inhibition. Toxicity of copper surfaces to 25 µL bacterial suspension is further evident from the approximately 2-log decrease in viable bacterial count after 60 min exposure (Fig. 3e,f). When 50 µL of Cu biosensor bacteria was added to copper coupons, Cu-dependent induction of bioluminescence was observed (Fig. 3a). However, the bioluminescence of constitutively bioluminescent *E*. *coli* strain in 50 µL decreased (Fig. 3b) which allowed us to conclude that the concentration of Cu ions released from copper coupons in this volume also exhibited a toxic effect which in Cu biosensor was masked by Cu-driven bioluminescence induction. Indeed, when the released concentration of Cu (11 µg per surface or 215 µg Cu/mL bacterial suspension) was compared with the copper standard curve, it was very close to toxic Cu concentrations (grey arrows in Fig. 3c,d). Differently from the 50 µL volume, no decrease in bioluminescence of the constitutively bioluminescent *E*. *coli* was observed when bacteria were applied to C11000 surfaces in 75 µL and also the bioluminescence induction of copper sensor strain in 75 µL was higher than in 50 µL volume. Additionally, the released concentration of copper ions in 75 µL (10 µg per surface or 130 µg/mL bacterial suspension) was lower than in 50 µL (Fig. 3c,d). Considering these results 75 µL volume was considered optimal for further analyses. Volumes larger than 75 µL/cm^2^ were not considered due to their instability on the surface.

#### Optimized protocol for the analysis of copper and copper alloy surfaces with bioluminescent bacteria

The optimized surface analysis protocol was tested on 1 × 1 cm coupons of a variety of copper and copper alloy surfaces (Table 1). The protocol included the placement of 1 × 1 cm coupons into 12-well microplates on 15 mm plastic adapters (Fig. 1a and Supplementary Figure S1), addition of 75 µL bacteria in LB medium at OD_600_ 0.15 (approximately 1 × 10^7^ cells/cm^2^) and direct measurement of bioluminescence during 60 min exposure at room temperature.

**Table 1:** Copper and copper alloy surfaces used. Specification of the alloys is per their Unified Numbering System (UNS) and according to Copper Development Association Inc.

UNS code	Trade name	Cu, %	Zn, %	Ni, %	Fe, %	Sn, %	Pb, %	Mn, %
C11000	Electrolytic Tough Pitch Copper	99.9	—	—	—	—	—	—
C51000	Phosphor Bronze, 5%	94.2	0.3	—	0.1	5.8	0.005	—
C70600	Copper-Nickel, 10%	89	—	10	1	—	—	—
n.a.	Nordic gold	89	5	—	—	1	—	—
C26000	Cartridge Brass, 70%	69–72	28–31	—	0.05	—	0.07	—
C71500	Copper-Nickel, 30%	64–69	1	29–33	0.4–1		0.05	1
C75200	Nickel Silver, 65–18	63–67	13–20	17–20	0.25		0.05	0.5
C28000	Muntz Metal, 60%	59–63	37–41		0.07		0.09	

“n.a.” – not available;“—” – not stated by manufacturer.

#### Constitutively bioluminescent E. coli for general toxicity assessment of surfaces

**Figure 4:**
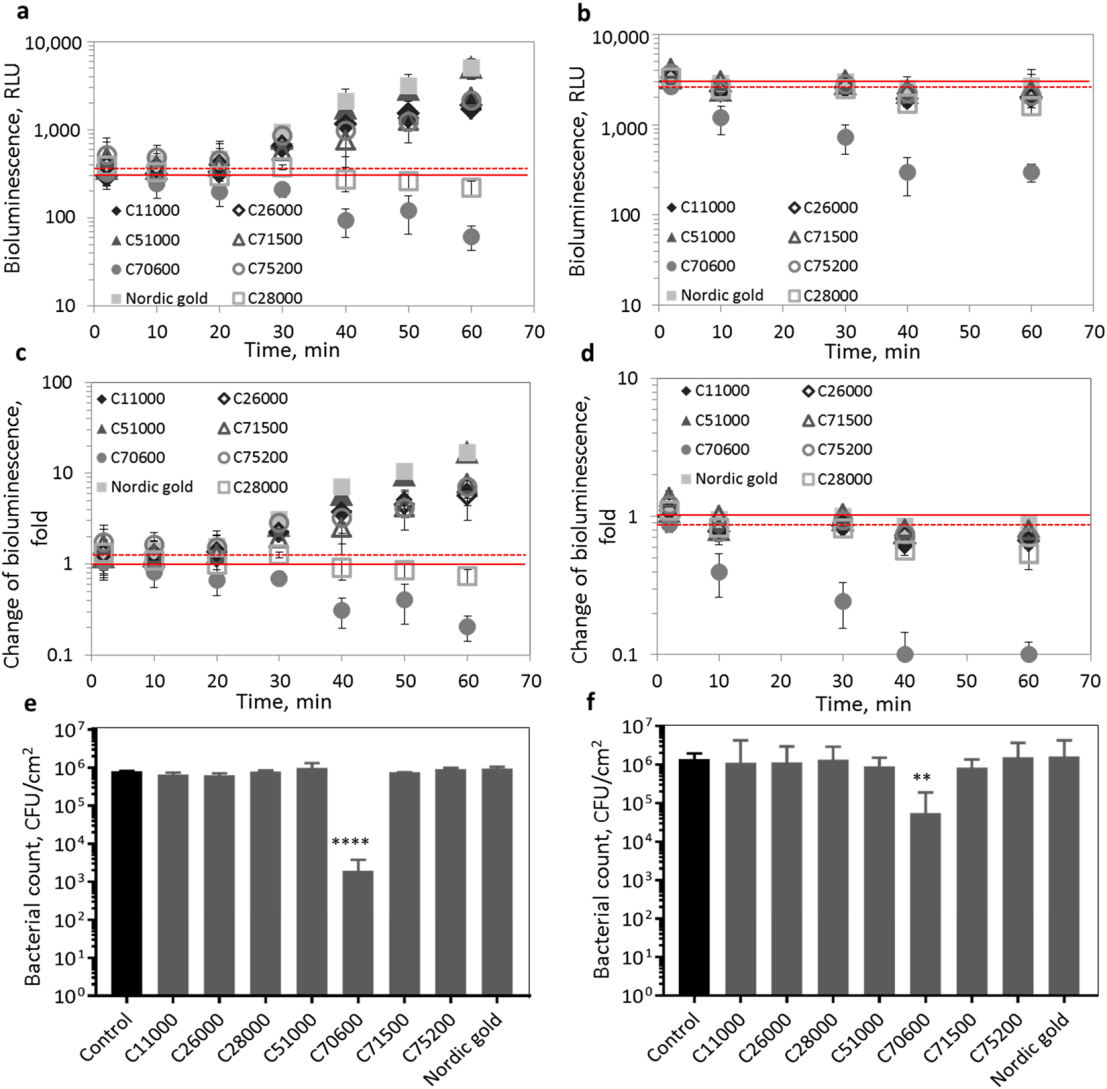
Changes of bacterial bioluminescence on copper and copper alloy surfaces. Results on Cu-induced *E*. *coli* (pSLcueR/pDNPcopAlux) (**a,c,e**) and on constitutively bioluminescent *E*. *coli* (**b,d,f**). Bioluminescence in relative light units (**a,b**) and fold change (**c,d**) when exposed to copper surfaces: C11000 (99.9% Cu), C51000 (94.2% Cu, 5.8% Sn), C70600 (89% Cu, 10% Zn), C26000 (69–72% Cu, 28–31% Zn), C71500 (64–69% Cu, 29–33% Ni), C75200 (63–67% Cu, 13–20% Zn,17–20% Ni), C28000 (59–63% Cu, 37–41% Zn) during 60 min in LB medium. Solid red line indicates background (non-exposed) bioluminescence and dotted red line significantly increased (Cu biosensor) or decreased (constitutively bioluminescent *E*. *coli*). (**e,f**) Viable bacterial counts on copper surfaces after 60 min exposure compared to control (PE) surface. Significant differences in viable counts from control value based one-way analysis of variance (ANOVA) and Dunnett’s multiple comparisons test are marked p < 0.1(.); p < 0.05(*); p < 0.01(**); p < 0.001(***); p < 0.0001(****).

One of the main advantages of the analysis protocol presented in this study is its rapidity: bioluminescent bacteria are applied onto the surfaces, and bioluminescence readings can be measured at any desired timepoint, starting from 20 min after exposure. The bioluminescence of constitutively bioluminescent *E*. *coli* (pDNlux) on copper and copper alloy surfaces shown in Fig. 4b demonstrates that the bacterial bioluminescence decreased on the tested surfaces to different extents indicating different toxicity of the studied surfaces. To verify that the decrease in bioluminescence was also correlated with decrease in viable bacterial count in the assay, we analyzed *E*. *coli* viability after 60 min exposure (Fig. 4f) and observed that the C70600 surface that caused a significant substantial decrease in bioluminescence caused also a significant decrease in viable bacterial counts. To further correlate bacterial bioluminescence and viability in our novel test system, we exposed the constitutively bioluminescent bacteria to different copper solutions, placed them onto control PE surfaces and measured both bioluminescence as well as registered viable counts. As shown on Supplementary Fig. S2, there was a clear correlation between bioluminescence and bacterial viability (r = 0.95, CI 95% 0.9–0.98; p < 0.0001), evaluated on the basis of traditional cultured plate counts. Based on linear regression between decrease of bioluminescence and viable counts (R^2^ = 0,91; y = 117620*x + 906854; Supplementary Fig. S2) we were able to calculate that e.g., 10% decrease in bioluminescence translated to the death of 2.1 × 10^6^ CFUs and 90% decrease in bioluminescence to the death of 1.1 × 10^7^ CFUs. Based on those data we suggest that screening of bacterial bioluminescence on solid surfaces could indeed be used as a rapid (60-min) tool for the evaluation of their antibacterial activity.

#### Analysis of copper ion release with the bioluminescent copper sensor

**Figure 5:**
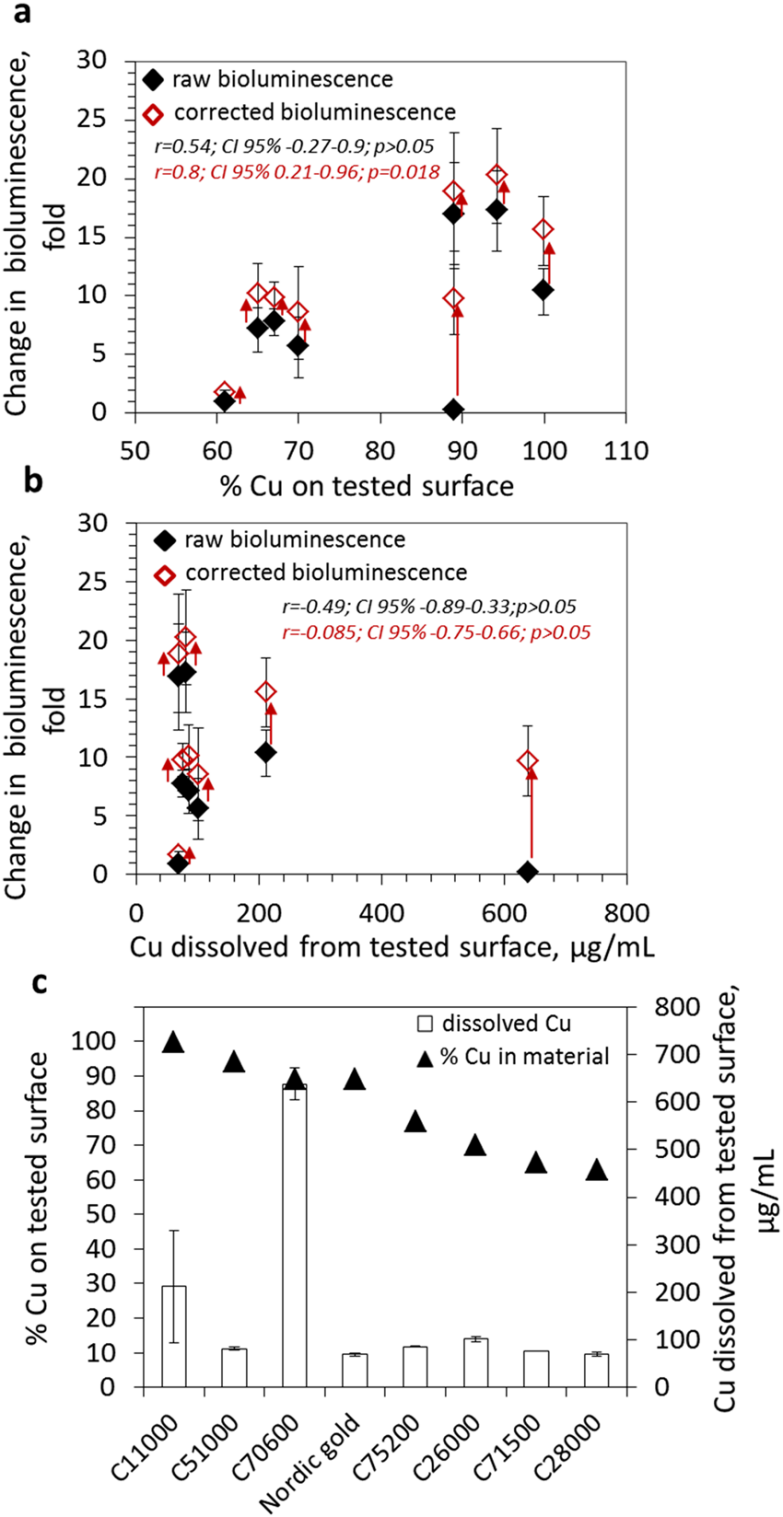
Correlation between Cu biosensor induction by copper surfaces and Cu content or dissolution of the surface material. Linear correlation between raw and corrected bioluminescence induction of the Cu biosensor on copper and copper alloy surfaces and Cu content of those surfaces (**a**) or copper release from these surfaces (**b**) after 60 min. Arrows indicate the difference between raw and corrected bioluminescence induction. All experiments were performed in LB medium in three replicates. (**c**) Copper content and release of Cu from the different copper surfaces in LB medium. Mean and standard deviation of three replicates are shown.

As already mentioned, surface released Cu ions have been considered as the main reason for antibacterial activity of copper-based surfaces and, therefore, addition of bioluminescent Cu sensor bacteria to the analysis with constitutively bioluminescent strains was considered relevant in evaluating the mechanism of antibacterial activity of copper-based surfaces. Results obtained with Cu biosensor *E*. *coli* (pSLcueR/pDNPcopAlux) on different copper and copper alloy surfaces are shown on Fig. 4. From eight tested surfaces, six induced the bioluminescence of the biosensor strain (Fig. 4a,c). Two surfaces that did not induce the biosensor, contained either the lowest amount of Cu (C28000, 63% Cu, Table 1) or were toxic and inhibited also the bioluminescence of the constitutively bioluminescent strain (C70600; see also decreased viable counts on that surface in Fig. 4e,f). In order to account for this toxicity-driven decrease of bacterial bioluminescence, the results of the constitutively bioluminescent *E*. *coli* strain were used to correct the bioluminescence induction of the Cu biosensor strain (see red symbols in Fig. 5). The corrected bioluminescence induction values of the Cu sensor strain correlated with Cu content of the surfaces (r = 0.8; CI 95% 0.21–0.96; p = 0.018, Fig. 5a) whereas there was no significant correlation between Cu content and raw bioluminescence data (r = 0.54; CI 95% −0.27–0.9; p > 0.05; Fig. 5a). However, there was also no significant correlation between Cu biosensor bioluminescence and Cu ion release from the surfaces during 60 min of biosensor assay (r = −0.49; CI 95% −0.89–0.33;p > 0.05 for raw values and r = −0.085; CI 95% −0.75–0.66; p > 0.05 for corrected values; Fig. 5b). Indeed, the TR-XRF measured dissolution of copper from different surfaces were not in line with surface Cu content (Fig. 5c) and, based on an earlier study by Suarez *et al*.^22^, we propose that in addition to surface chemical composition also certain physical properties of the surfaces such as roughness affect the release of metals from solid surfaces. We suggest that bioavailability of Cu on copper-based surfaces to bacteria was affected by direct contact between bacterial cells and the surfaces followed by local release of copper in the bacterial microenvironment. A similar observation has been reported by us in earlier papers for soil samples^23,24^. In this respect, bacterial biosensors can be used as unique tools to analyze surface-released bioavailable metal ions directly on solid samples.

## Discussion

Here we present a novel, robust and rapid test method that can be applied for the analysis of solid surfaces which in this study was optimized for copper and copper alloy-based surfaces. The method allows direct analysis of the surfaces and does not require an additional cultivation step for colony counting or other indirect measurement. The test protocol requires the use of bioluminescent bacteria that are applied to the surface in liquid form, exposure for a specified period at room temperature and real-time measurement of bioluminescence. Although a variety of natural as well as recombinant bioluminescent bacteria can be obtained through commercial suppliers or from research laboratories are available, we here used two *E*. *coli* strains because this bacterium is a medically relevant test organism and because we have constructed a series of *E*. *coli-*based bioluminescent sensor strains that enable detection of metal ions^25^ as well as other intracellular stress conditions^26^. A liquid test method was chosen for several reasons, the most important being the fact that bacterial bioluminescence is not produced in dry conditions but also because the results of dry tests have shown to greatly depend on different testing conditions, e.g., temperature and relative humidity. Here we identified that inoculum volume on copper coupons as well as the exposure medium had a significant effect on antibacterial efficacy of the surfaces. Our results showed that a poor medium, e.g., 1:500 diluted NB medium which is a suggested medium in ISO 22196 standard test protocol for solid surfaces, is not suitable when bioluminescent bacteria are used as the nutrient supply in poor media is too low to support the energy-expensive bioluminescence reaction. The latter can be only supported by organic-rich media, e.g., LB medium which is a standard microbiological growth medium, or TSB medium which is a suggested medium in US EPA standards for the testing of copper surfaces^12,13^. Similar exposure medium-dependent antibacterial effects of copper surfaces have been observed earlier e.g., by Molteni *et al*.^20^ who demonstrated that exposure to medium containing Tris buffer showed lower antibacterial effect than for example phosphate buffer and Hans *et al*.^19^ who showed 10–50-fold faster copper ion release in Tris-HCl buffer compared to phosphate buffered saline (PBS). Indeed, as also demonstrated by us, the amount of released Cu ions in the different exposure media differed around 50-fold, being the lowest in 1:500 diluted NB medium (0.7 µg Cu per cm^2^ surface; Fig. 2c) and the highest in Heavy Metal MOPS medium supplemented with 0.5% cas-aminoacids (53 µg Cu per cm^2^ surface; Fig. 2c). At the same time, in the same volume of MillIQ water the released Cu concentration was 0.3 µg Cu per cm^2^ surface. Therefore, certain media components, most notably organics, significantly enhanced the release of Cu ions from copper surfaces. As a compromise between supporting bacterial bioluminescence production and release of Cu ions from the surfaces in the range that is capable of inducing the bioluminescence of Cu-sensing bacteria, LB was selected as an optimal medium for further testing. Yet as our test optimization indicated, the volume of bacterial suspension in LB medium also affected the release of ionic copper from the tested surfaces. A smaller volume (25 µL) of bacterial suspension on surfaces released significantly more copper from the surface than a larger volume (75 µL), thus inhibiting bacterial bioluminescence and interfering with Cu biosensor measurement. This finding suggested that a larger volume, in our case 75 µL bacterial suspension on the surface, supports bacterial bioluminescence production and is favorable.

One of the novelties of our test system is the possibility to use bacteria that report on the mechanisms of antibacterial actions of the surfaces. In our previous studies we have constructed a range of bioluminescent bacteria that not only are able to report on the presence of bioavailable metal ions in their environment^25^ but also bacteria whose bioluminescence is induced by e.g., the presence of reactive oxygen species and DNA damage^26^. As it is generally suggested that the key mechanism through which copper surfaces exhibit their antimicrobial activity is the release of copper ions^4,17,20,27^, in this study we applied the copper sensing bioluminescent *E*. *coli* strain to analyze and quantify the release of bioavailable ionic copper from the studied surfaces. However, as also other toxicity pathways, e.g., oxidative damage in the form of lipid peroxidation and oxidation of proteins due to superoxide and hydroxyl radicals, and DNA degradation have been proposed for copper surfaces^3,5,28–31^, other previously mentioned mechanism-based sensor bacteria could be used in this test format. Our results with bioluminescent copper sensors showed that significant amounts of Cu ions were released and entered bacterial cells from copper and copper alloy-based surfaces and that this released and bacteria-bioavailable fraction showed a positive correlation with copper content on the surfaces. Interestingly, there was no significant correlation between surface released Cu and response of the copper biosensor: on six surfaces that released 69 to 85 µg Cu /mL, the induction of the copper biosensor varied from 1 to about 20-fold. The fact that Cu biosensor induction correlated better with material properties (i.e., Cu content) than with material-released Cu suggests that bacteria-bioavailable Cu is rather a function of direct interactions between microbes and surfaces than of simple Cu dissolution. These direct interactions could lead to variable local Cu ion concentrations in bacterial microenvironments. Similar observations have been reported previously by us for soil samples where bioavailability of Cu and also other bivalent metals (e.g., Cd and Pb) had relatively poor correlation with water-extracted metal ions^23,24^. In the case of surfaces, similar direct contact-driven bacterial effects have been observed by Zeiger *et al*.^27^ who demonstrated that the physical nature of copper surfaces affected their antibacterial activity. These direct interactions between microbes and solid surfaces can be only elucidated using real-time biological analysis methods, as the one described here. Considering this unique capability of our test system and the rapidity and simplicity of the analysis procedure we propose that direct analysis of bacterial bioluminescence from solid surfaces could be used as an efficient screening tool to evaluate the bioavailability of Cu (and in case of other types of metal surfaces, other metals, e.g., release of Ag ions from antibacterial silver nanoparticles^32^) from antibacterial copper-based surfaces, and their toxicity. As with any (anti)bacterial assay, our protocol involves pre-cultivation of bacteria, but lacks the need for cell removal (and associated issues regarding removal efficiency versus survivor viability) as well as post-cultivation for viable cell count determination or microscopy based counting steps. Results are collected in real-time and in digital form that is easy to automate. Given the good correlation between the survival of bacteria on copper-based surfaces and the bioluminescence of constitutively bioluminescent bacteria on the surfaces, our suggestion for the use of the presented direct surface measurement set-up is justified.

In conclusion, in this study we have presented a protocol for the direct analysis of Cu surfaces for i) their bioavailable copper release and ii) antibacterial efficacy using a Cu ion inducible bioluminescent *E*. *coli* strain and a constitutively bioluminescent *E*. *coli* strain. The main advantages of our test protocol are: i) rapidity (an indication on antimicrobial effects can be obtained within 30–60 min), ii) cost efficiency (due to saving of laboratory consumables needed for traditional colony counting), and iii) possibility to in addition to antimicrobial efficacy evaluate its cause. Although our methodology was optimized for copper-based surfaces and *E*. *coli* bacteria in this study, it’s use can certainly be extended to other metallic surfaces, including but not limited to, silver and zinc-based surfaces and other types of bioluminescent bacteria.

## Methods

### Surfaces and their preparation for test

The copper-based surfaces tested here (Table 1) originated from Copper Development Association Inc. and were cut to 1 × 1 cm coupons. Prior to experiments, all copper alloy coupons were treated by washing with acetone and ethanol using glass beads. Each coupon was individually flamed for sterilization purposes prior to inoculation. As control surfaces, 1 × 1 cm squares were cut from polyethylene (PE) plastic, sterilized with ethanol, and allowed to dry.

### Preparation of bacteria for the test

Recombinant bioluminescent strains of *E*. *coli*, a constitutively bioluminescent strain *E*. *coli* (pDNlux)^25^, and a Cu-induced bioluminescent strain *E*. *coli* (pSLcueR/pDNPcopAlux)^25^ were used. The bacteria were routinely maintained on LB agar (LB medium (Supplementary Table 1) with 15 g agar/L) supplemented with 10 µg/mL tetracycline (*E*. *coli* (pDNlux)) or 10 µg/mL tetracycline and 100 µg/mL ampicillin (*E*. *coli* (pSLcueR/pDNPcopAlux)). In preparation for the test, the bacteria were grown in the appropriate medium overnight, diluted 1:50 with fresh medium and grown until OD_600_ 0.6. Then the cells were diluted either with LB medium until OD_600_ 0.15 or washed and diluted in a different test medium (see below).

### Optimization of the test set-up

Testing of surfaces with recombinant bioluminescent bacteria was optimized for the black 12-well microplate configuration and room temperature (20–22 °C). This plate type was selected to accommodate 1 × 1 cm sized coupons and respective control surfaces inside the wells. However, depending on the size of specific surfaces, microplates with less (e.g., 6-well plate) or more (e.g., 24-well plate) wells can be selected. For test optimization, the 1 × 1 cm PE plastic surfaces on black 12-well microplates (Cellvis, CA, US) were placed onto the well bottoms or raised by using 5, 12 and 15 mm high plastic adaptors (Fig. 1), to bring the surfaces closer to photomultiplier tube and increase the bioluminescence signal intensity. A 75 µL drop of *E*. *coli* (pSLcueR/pDNPcopAlux) culture (OD_600_ 0.15) in LB medium containing ~1 × 10^7^ cells was placed onto the surfaces on 12-well microplates and spread so that the coupon surface was covered. The relative luminescence (RLU) was measured from the middle of each well using a Promega Glowmax plate luminometer or Fluoroskan Ascent plate luminometer. For setting the background value, the luminescence signals of empty wells were measured; in bacterial experiments only signals that exceeded this background at least 3-fold were counted. Using 15 mm high adaptors on 12-well plates was considered as the most suitable test format for further experiments.

To validate the performance of bacterial bioluminescence measurement on surfaces and in 12-well plates, bioluminescence of *E*. *coli* (pSLcueR/pDNPcopAlux) in 12- and 96-well plates was compared; 96-well plates were used for comparison as an already established method used by our and other research groups in a variety of reports^25,33,34^. To compare exactly the same conditions in 12- and 96-well plates, 150 µL of *E*. *coli* (pSLcueR/pDNPcopAlux) bacteria (OD_600_ 0.15 in LB medium) were mixed with variable (0.1–100 µg Cu/mL) concentrations of CuSO_4_ × 5H_2_O (Sigma-Aldrich) (0.002 to 2000 µg/mL Cu); 75 µL of the mixture was added to PE control surface on 15 mm adaptor in a 12-well plate and 75 µL of the mixture was pipetted to a well of a black 96-well microplate. The bioluminescence of both plates was measured after 60 min incubation at room temperature and the readings from 96 and 12-well plates were correlated. Three independent experiments were performed.

To optimize the test medium, *E*. *coli* (pSLcueR/pDNPcopAlux) and *E*. *coli* (pDNlux) were suspended in LB which is a traditional bacterial growth medium, TSB (tryptic soy broth) that is one of the suggested media in US EPA suggested surface testing methods^12,13^, HMM (heavy metal MOPS medium) that was suggested for transition metal testing by LaRossa *et al*.^21^ and has been used earlier to study metal bioavailability to bacteria^18,25,35^ (in our experiments, HMM medium was supplemented with different amounts, 0.01 and 0.5% of casein amino acids), and 1:500 diluted NB (nutrient broth) that is suggested as an exposure medium in ISO 22196^14^ (for the composition of test media, see Supplementary Table 1). Prior to the test, bacteria pre-grown in LB until OD_600_ 0.6 were double washed (centrifugation at 5000 g for 5 min and resuspension in specific test medium) and diluted to OD_600_ of 0.15 using specific test medium. 75 µL of bacteria was spread onto C11000 surfaces on 15 mm high adaptors in 12-well plates, incubated for 60 min and bioluminescence was measured. Bacteria in each test medium were analyzed thrice. According to the results, LB and TSB were considered as most optimal and LB was used in all further experiments. In order to check for culture viability in different media after 60 min incubation on C11000 surfaces, the exposed bacteria were collected, serially diluted and plated onto NB (15 g agar/L) plates. Colonies were counted after 24–36 h incubation at 30 °C. To quantify release (dissolution) of copper from C11000, bacterial suspensions exposed to the surfaces in different media were collected, 1 mg/L Ga (internal standard) was added and Cu content was quantified using total reflection X-ray fluorescence (TR-XRF; S2 Picofox, Bruker).

Due to different volume requirements for the bacterial inoculum in different surface testing standards (5–20 µL on 2.5 × 2.5 cm surfaces followed by drying in US EPA^12,13^, 50 µL on 2 cm diameter surfaces followed by drying in EN13697^15^, 100 µL on 4 × 4 cm surfaces in ISO 22196 standard^14^), we here optimized the volume of bioluminescent bacteria on 1 × 1 cm surfaces. For that, 25, 50 and 75 µL of *E*. *coli* (pSLcueR/pDNPcopAlux) and *E*. *coli* (pDNlux) suspension in LB (25 µL being the smallest volume that was possible to be spread on the surface without visible drying during 60 min exposure and 75 µL being close to the maximum volume of droplet that remained stable on the surfaces while handling) was applied onto the 1 × 1 cm surfaces. In each exposure, the bacterial number was kept similar (~1 × 10^7^ cells per surface) and thus, 75 µL of bacteria was added at OD_600_ 0.15, 50 µL at OD_600_ 0.22 and 25 µL at OD_600_ 0.45. Bacteria were spread on C11000 coupons and bioluminescence was registered over 60 minutes. The experiment was performed in triplicate. In order to check for bacterial viability in different volumes after 60 min exposure on copper surfaces, the exposed bacteria were collected, diluted and plated onto NB agar plates. Colonies were counted after 24–36 h incubation at 30 °C and surface released (dissolved) copper was quantified using TR-XRF, as described above. According to the results, 75 µL was selected as optimal volume of bacteria on the surfaces.

### Analysis of copper and copper alloy surfaces for bacterial toxicity and bioavailable Cu

After optimization, copper and copper alloy coupons (Table 1) along with similar sized (1 × 1 cm) control surfaces (PE) were analyzed with constitutively bioluminescent *E*. *coli* (pDNlux) and Cu-induced *E*. *coli* (pSLcueR/pDNPcopAlux) bacteria (75 µL of bacterial suspension at OD_600_ 0.15 in LB medium spread on a surface) and bioluminescence of the bacteria was registered during 60 min. The results for copper surfaces (i.e., test surfaces) were expressed both in relative bioluminescence units (RLU) as well as in fold change in bioluminescence. Fold induction of the Cu sensor or constitutively bioluminescent *E*. *coli* was calculated as follows:

1 $${\rm{Fold}}\,{\rm{change}}\,{\rm{in}}\,{\rm{biolumin}}e{\rm{scence}}=\frac{{\mathrm{RLU}}_{{\rm{test}}{\rm{surface}}}}{{{\rm{RLU}}}_{{\rm{control}}{\rm{surface}}}}$$

In order to account for potential toxicity and unspecific effects of the tested surfaces, bioluminescence of the Cu biosensor strain prior to calculating the fold change (eq. 1) was corrected using *E*. *coli* (pDNlux):

2 $${\rm{Corrected}}\,{\rm{RLU}}\,{\rm{Cu}}\,{{\rm{sensor}}}_{\mathrm{test}\mathrm{surface}}={\rm{RLU}}\,{\rm{Cu}}\,{{\rm{sensor}}}_{\mathrm{test}\mathrm{surface}}\times \frac{{\rm{RLU}}\,{\rm{constitutive}}\,{{\rm{strain}}}_{\mathrm{control}\mathrm{surface}}}{{\rm{RLU}}\,{\rm{constitutive}}\,{{\rm{strain}}}_{\mathrm{test}\mathrm{surface}}}$$

To check for bacterial viability of Cu biosensor and constitutively bioluminescent bacteria on the different copper surfaces after 60 min exposure, the exposed bacteria were collected, diluted and plated onto NB agar plates. Colonies were counted after 24–36 h incubation at 30 °C and surface-released (dissolved) copper was quantified using TR-XRF as described above. Additionally, to correlate the decrease of bioluminescence and colonies of the constitutively bioluminescent *E*. *coli* on surfaces, *E*. *coli* (pDNlux) was exposed to CuSO_4_ solutions (100–1000 µg Cu^2+^/mL) for 60 min after which bioluminescence and CFU count on nutrient agar plates was registered.

### Statistical analysis

Mean values, standard deviations and correlation plots were produced by Microsoft Excel standard functions. Correlation statistics, linear regression and analysis of variance (ANOVA) followed by Dunnett’s or Bonferroni’s multiple comparisons test were executed in GraphPad Prism 7.04. Alpha value 0.05 and two-tailed calculations were used where applicable.

### Data availability

The datasets generated during and/or analysed during the current study are available from the corresponding author on reasonable request.

## Electronic supplementary material

Supplementary material
