# Supplementary material for: Rapid in situ assessment of Cu-ion mediated effects and antibacterial efficacy of copper surfaces
Source: Sci Rep. 2018 May 25;8:8172. doi: 10.1038/s41598-018-26391-8 (PMC5970231; doi:10.1038/s41598-018-26391-8)
Supplement: Supplementary file 1 — Supplementary material [file 41598_2018_26391_MOESM1_ESM.docx]

**Supplementary Material for**

**Rapid *in situ* assessment of Cu-ion mediated effects and antibacterial efficacy of copper surfaces**

Merilin Rosenberg^1,2^, Heiki Vija^1^, Anne Kahru^1,3^, C. William Keevil^4^*, Angela Ivask^1^*

^1^ Laboratory of Environmental Toxicology, National Institute of Chemical Physics and Biophysics, Tallinn, Estonia

^2^ Department of Natural Sciences, Tallinn University of Technology, Tallinn, Estonia

^3^ Estonian Academy of Sciences, Kohtu 6, Tallinn, Estonia

^4^ Faculty of Natural and Environmental Sciences, Centre for Biological Sciences, University of Southampton, Southampton, UK

* Corresponding authors: C.W.Keevil@soton.ac.uk, angela.ivask@kbfi.ee

**Table S1. Composition of bacterial test media**

| **Test medium** | | **Composition** | **pH** | **Characteristics** | **Reference** |
| --- | --- | --- | --- | --- | --- |
| LB | Luria-Bertani | *per* L:  10g tryptone  5 g yeast extract  5 g NaCl | 7.2 | Traditonal bacterial growth medium | Sigma-Aldrich |
| TSB | Tryptic Soy Broth | *per* L:  17 g casein peptone  3 g soy peptone  5 g NaCl  2.5 g K_2_HPO_4_  2.5 g glucose | 7.3 | Suggested medium in US EPA standards ^1, 2^ | Sigma-Aldrich |
| HMM (0.5% AA) | Heavy Metal MOPS Medium, supplemented with 0.5% aminoacids | *per* L:  0.63 g MOPS  0.28 g KCl  0.04 g NH_4_Cl  0.009 g MgSO_4_  0.4 g glucose  0.002 g glycerol-2-phosphate  0.005 mg FeCl_3_  5 g casein aminoacids | 7.2 | Used for metal testing in earlier studies ^3-5^ | LaRossa *et al*. ^6^ |
| HMM (0.01% AA) | Heavy Metal MOPS Medium, supplemented with 0.5% aminoacids | *per* L:  0.63 g MOPS  0.28 g KCl  0.04 g NH_4_Cl  0.009 g MgSO_4_  0.4 g glucose  0.002 g glycerol-2-phosphate  0.005 mg FeCl_3_  0.1 g casein aminoacids | 7.2 | Used for metal testing in earlier studies ^3-5^ | LaRossa *et al*. ^6^ |
| NB | Nutrient Broth | *per* L:  10 g peptone  3 g meat extract  5 g NaCl | 6.8-7.2 | 500-fold dilution in water suggested as an exposure medium in ISO 22196 ^7^ | ISO 22196:2011^7^ |

**
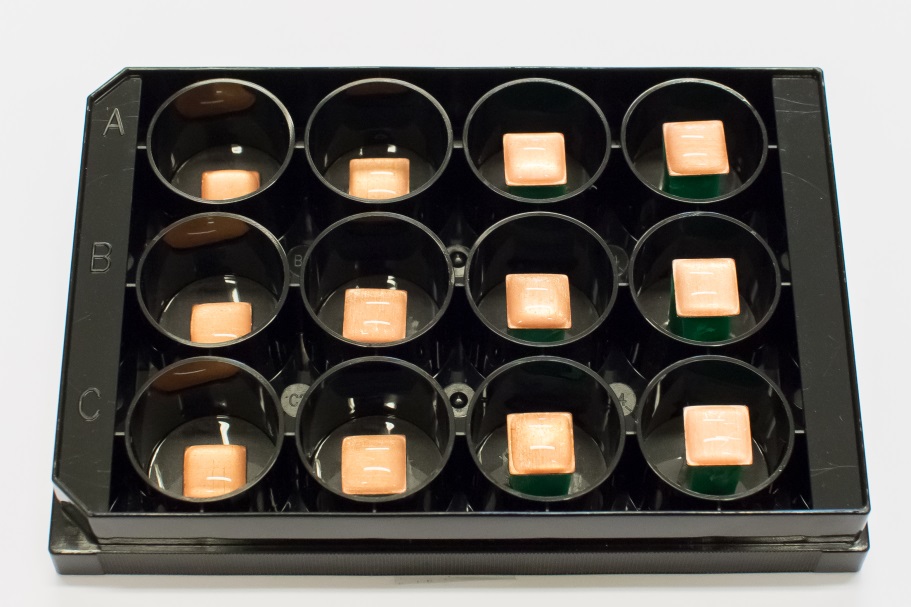
**

**Figure S1. Set-up of 12-well microplate for optimal signal collection from bioluminescent bacteria on 1×1 cm copper surfaces.** Photograph of a 12-well microplate in which the left column represents copper coupons on the plate bottom; left and right middle columns show coupons raised using 5 and 12 mm adapters, respectively; and the right column shows adapters raised using 15 mm plastic adapters; 75 µL bacterial suspension was applied to each copper coupon.

**
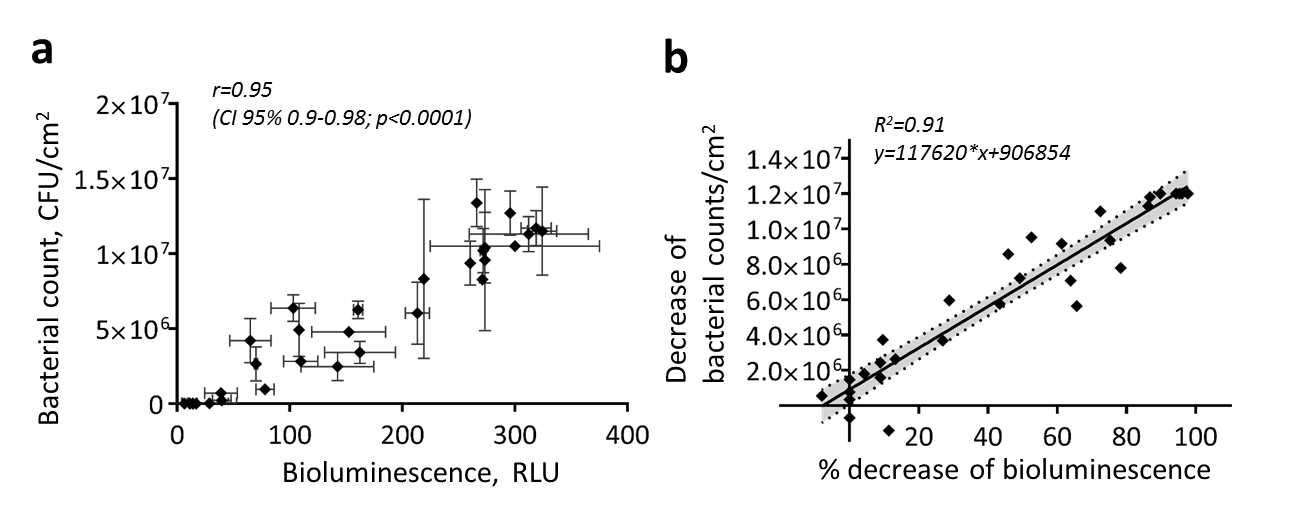
**

**Figure S2.** **Correlation between bioluminescence and viable counts of the constitutively bioluminescent *E. coli* strain on solid surfaces.** Correlation between bioluminescence and viable count of *E. coli* (pDNlux) that was exposed to CuSO_4_ solutions (100-1000 µg Cu^2+^/mL) of different toxicities on PE control surfaces for 30-60 min. (a) linear correlation between bioluminescence and colony counting method; every data point is a mean of two parallel experiments with standard deviation. (b) decrease of bacterial counts at different levels of bioluminescence inhibition; coefficient of determination, equation for linear regression and 95% confidence intervals (grey area) are shown. The linear regression equation enables calculation of the amount of colony forming units that are affected when certain decrease in bioluminescence is measured.

**References**

1. US Environmental Protection Agency. Test Method for the Continuous Reduction of Bacterial Contamination on Copper Alloy Surfaces. (2009)

2. US Environmental Protection Agency. Test Method for Efficacy of Copper Alloy Surfaces as a Sanitizer. (2009)

3. Ivask, A., Rõlova, T.& Kahru, A. A suite of recombinant luminescent bacterial strains for the quantification of bioavailable heavy metals and toxicity testing. *BMC Biotechnol* **9**, 41 (2009)

4. Käkinen, A., Bondarenko, O., Ivask, A.& Kahru, A. The Effect of Composition of Different Ecotoxicological Test Media on Free and Bioavailable Copper from CuSO_4_ and CuO Nanoparticles: Comparative Evidence from a Cu-Selective Electrode and a Cu-Biosensor. *Sensors* **11**, 10502 (2011)

5. Leedjärv, A., Ivask, A.& Virta, M. Interplay of Different Transporters in the Mediation of Divalent Heavy Metal Resistance in *Pseudomonas putida* KT2440. *J Bacteriol* **190**, 2680-2689 (2008)

6. LaRossa, R. A., Smulski, D. R.& Van Dyk, T. K. Interaction of lead nitrate and cadmium chloride with *Escherichia coli* K-12 and *Salmonella typhimurium* global regulatory mutants. *J Ind Microbiol* **14**, 252-258 (1995)

7. International Organization for Standardization. ISO 22196:2011. Measurement of antibacterial activity on plastics and other non-porous surfaces. (2011)
